# Retroperitoneal laparoscopic pyelolithotomy versus extra corporeal shock-wave lithotripsy for management of renal stones

**DOI:** 10.4103/0972-9941.72596

**Published:** 2010

**Authors:** Jagdish Chander, Nikhil Gupta, Pawanindra Lal, Pawan Lal, Vinod K Ramteke

**Affiliations:** Department of Surgery, Maulana Azad Medical College, New Delhi - 110 002, India

**Keywords:** Laparoscopic pyelolithotomy, minimal invasive, renal stones, shock waves, shock wave lithotripsy

## Abstract

**AIM:**

The purpose of this study was to evaluate the role of retroperitoneal laparoscopic pyelolithotomy (RPPL) and its comparison with extra corporeal shock wave lithotripsy in the management of renal calculi.

**MATERIALS AND METHODS:**

The study was carried out in the Department of surgery, Maulana Azad Medical College, New Delhi, India. The study included 86 cases of solitary renal calculi in the retroperitoneoscopic (RPPL) group and 82 cases in the shock wave lithotripsy (SWL) group. The parameters compared were stone clearance, hospital stay, number of postoperative visits, mean time to resume normal activities, number of man days lost, and analgesic requirement.

**RESULTS:**

The RPPL group showed better stone clearance, fewer hospital visits, low analgesic requirement, fewer number of man days lost, and early resumption of normal activities, as compared to the SWL group.

**CONCLUSIONS:**

Shock wave lithotripsy, being a noninvasive modality, is an established procedure all over the world. However RPPL achieves comparable or better results in high volume centers.

## INTRODUCTION

The management of renal stones has changed in the last two decades from invasive to minimally invasive to non-invasive procedures, with the advent of percutaneous nephrolithotripsy (PCNL), extracorporeal shock wave lithotripsy (ESWL), and so on. The advances made in the retroperitoneoscopic management of renal diseases, including renal stone disease, has added a new dimension to the already available techniques. The role of retroperitoneoscopic surgery is established for the removal of nonfunctioning kidneys and renal tumors.[1] However, the role of RPPL in management of renal stones is yet to be established. Successful laparoscopic retroperitoneal management of renal stones has been described,[2–11] although the indications have not been defined and outcomes not compared with the established techniques such as SWL and PCNL. This study is an endeavor to evaluate the role of RPPL in the management of renal calculi and to compare it with ESWL for renal stones.

## MATERIALS AND METHODS

The study was conducted in the Department of surgery, Maulana Azad Medical College and the associated Lok Nayak Hospital, New Delhi, India. It was approved by ethical committee of the institution. The study included 86 cases of solitary renal calculus in the retroperitoneoscopic (RPPL) group and 82 cases in the SWL group. Informed written consent was taken from all the patients. There were eight patients with caliceal calculi in the RPPL group, while the SWL group had seven patients with caliceal calculi. The study was prospective and randomised. Both groups were age- and sex- matched. The preoperative assessment of all the patients included the following investigations: plain X-ray of the KUB region, kidney function tests, namely, blood urea and serum creatinine, urine routine and microscopy, urine culture and antibiotic sensitivity, intravenous urography, ultrasound KUB, and renal scan whenever indicated. The patients with urinary infection received a course of antimicrobial therapy and they were taken up for the procedure after the urine culture was sterile.

The mean stone size was less than or equal to 2.5 cm in both the groups. The parameters compared were stone clearance, hospital stay, number of hospital visits, mean time to resume normal activities, number of man days lost, and the analgesic requirement. The definition of stone clearance was the absence of any radio opaque shadow on plain X-ray KUB. Statistical analysis was done using the SPSS software version 12 (SPSS Inc. Illinois, USA).

The SWL was carried out with the Siemens Lithostar electromagnetic shockwave system, while the RPPL was carried out with the available laparoscopic instruments. SWL was carried out on an out patient basis. A double J ureteral stent was inserted in all the patients before the start of lithotripsy, for the sake of uniformity in both arms of the study. The procedure was carried out under analgesia (Diclofenac 75mg, intramuscular) with 3000 shock waves given to the patient per sitting. The patients were evaluated two weeks after each SWL treatment with X-ray KUB (AP view) to look for stone clearance. No significant change in stone size after four SWL treatments was taken as failure of the procedure. The patients were discharged on analgesics and antibiotics immediately after the SWL procedure.

All patients of the RPPL group had a double ‘J’ ureteral stent inserted on the side of the surgery preoperatively, under local anesthesia. The first port was inserted through a 15 mm incision made just below the tip of the twelfth rib. The muscles were divided under vision; the dorsolumbar fascia was incised and the retroperitoneal space was entered. Blunt finger dissection was carried out posterior to the kidney. Wherever possible the Gerota’s fascia was either incised under vision or breached with the finger tip. The peritoneal dissection balloon (PDB) was inserted and inflated with air (approximately 500 ml) inside the Gerota’s fascia. The balloon was kept inflated for 3-5 minutes to achieve hemostasis. In cases where it was not possible to inflate the balloon inside the Gerota’s fascia, the balloon was inflated posterior to the kidney and a 0° telescope was introduced through this port and the peritoneum was further stripped off the transversalis fascia with counter pressure from the opposite side.

The second 10 mm port was placed in the line of the first port, just above the iliac crest. The third 5 mm port was placed anteriorly, midway between the first two ports, in such a manner that the three ports formed an equilateral triangle. The fourth port was placed posteriorly in the line of the third port just lateral to the Quadratus lumborum. Secondary ports were inserted with finger guidance. The laparoscope was introduced through the second port. A 5 mm triflange retractor introduced through the first port was used to retract the kidney anteriorly. Dissection was carried out through the third or fourth port. We preferred to approach the pelvis of the kidney directly, however in cases where the pelvis was either not easily accessible or there was an aberrant vessel, the upper end of the ureter was exposed and it was traced up to the pelvis. The pelvis was dissected and a pyelotomy incision was made with a hook dissector using monopolar cautery. The Gil-Vernet’s plane was dissected wherever necessary, to carry out extended pyelolithotomy. The Gil-Vernet’s plane dissection has to be done in case of intrarenal pelvis. In fact, once one overcomes the learning curve of this procedure, it is easier to dissect the Gil-Vernet’s plane compared to open surgery, as laparoscopy offers direct vision of the plane of dissection. It is not unusual to find that one ends up dissecting this plane more often and inadvertently as it is easier to do in RPPL.

A longitudinal pyelotomy incision was made in patients of calculus in renal pelvis; however, in cases of extended pyelolithomy, a curvilinear pyelotomy incision was made, with an extension of the incision into the appropriate calyx. After extraction of the calculus, the pyelotomy incision was closed meticulously with a 3-0 vicryl. The operated area was drained with a tube drain and the wound closed. The drain was removed when the drainage was less than 25 ml/day. The double ‘J’ stent was removed on an out patient basis. Post operatively, an X-ray of the KUB was carried out after two weeks, to confirm stone clearance.

## RESULTS

The results between SWL and RPPL were compared considering the following parameters: age distribution, stone size, stone clearance, time taken to resume normal activities, total requirement of analgesics, number of hospital visits, and complications.

The average age between the two groups was comparable. The average age of the SWL group was 36.16 ± 11.0 years, whereas, that of the RPPL group was 36.47 ± 11.38 years. Both Levene’s and the t-test showed an insignificant *P* value. Both groups were matched for gender.

The average stone size in SWL was 1.021± 0.36 cm (range 0.8-2.3 cm) and in the RPPL group it was 1.730 ± 0.53 cm (range 1.1-2.5 cm). Both Levene’s and t-test showed the difference in stone size to be significant (*P* value < 0.001). The average stone size was larger in the RPPL group as compared to the SWL group.

There were eight patients with caliceal calculi in the RPPL group, while the SWL group had seven patients with caliceal calculi. Stone clearance was achieved in all the cases of the RPPL group barring three patients (3.5%) with caliceal calculi which were converted to an open procedure [Table 1]. Average SWL treatment requirement was 2.3, which was comparable to the data available in literature.[11] There were 13 failures (15.8%) in the SWL group. By the fisher exact test *P* < 0.05. Hence, there was a significant difference between the stone clearance rates of the two procedures.

**Table 1:** Stone clearance in RPPL and ESWL groups

			Group		Total
			ESWL	Retroperitone Oscopic Pyelolithotomy	
Stone clearance	No	Count	13	3	16
		% within GROUP	15.8%	3.5%	9.5%
	Yes	Count	69	83	152
		% within GROUP	84.2%	96.5%	90.5%
Total		Count	82	86	168
		% within GROUP	100.0%	100.0%	100.0%

Post operative stay in the RPPL group ranged from 2 to 10 days with a mean of 3.12 days.

Time taken to resume normal activities in the RPPL group was 1.09 ± 0.29 days and it was 2.37 ± 0.69 days in the SWL group. This difference was statistically significant, *P* < 0.001 [Table 2]. The time taken to resume normal activities was more in the SWL group.

**Table 2:** Time taken to resume normal activities

	Group	N	Mean	Std. Deviation	Std. Error Mean
Time to resume normal activities (days)	Retroperitoneoscopic Pyelolothotomy	86	1.09	0.292	0.032
	ESWL	82	2.37	0.694	0.077

The analgesic requirement was 889 ± 396 mg of Diclofenac sodium in the SWL group and 102.8 ± 47.7 mg in the RPPL group. Analgesic requirement was far less in the RPPL group as compared to the SWL group, and this difference was statistically significant (*P* < 0.001) [Table 3]

**Table 3:** Analgesic requirement, hospital visits, and blood loss

Group		Analgesic req.(mg)	Blood loss (ml)	No. of hospital visits
ESWL	N	82		82
	Minimum	450		2
	Maximum	2700		14
	Range	2250		12
	Mean	889.02		4.84
	Std. Deviation	395.969		2.857
	Median	800.00		4.00
	Std. Error of Mean	43.727		0.315
Retroperitoneoscopic Pyelolithotomy	N	86	86	86
	Minimum	50	10	2
	Maximum	300	60	3
	Range	250	50	1
	Mean	102.81	23.49	2.53
	Std. Deviation	47.732	9.427	0.502
	Median	100.00	20.00	3.00
	Std. Error of Mean	5.337	1.017	054
Total	N	168	86	168
	Minimum	50	10	2
	Maximum	2700	60	14
	Range	2650	50	12
	Mean	500.77	23.49	3.66
	Std. Deviation	485.252	9.427	2.329
	Median	450.00	20.00	3.00
	Std. Error of Mean	38.125	1.017	0.180

In the SWL group, the average clinic visit per patient was 4.8 ± 2.8, whereas, in the RPPL group, it was 2.5 ± 0.5. Blood loss in the RPPL group was 23.5 ± 9.4 ml. Blood loss could not be estimated in the SWL group. Clinic visits were fewer in the RPPL group as compared to the SWL group (*P* < 0.001).

The complications encountered in the RPPL group were peritoneal rent in five, although this did not require conversion, wound infection in two and prolonged drainage (72 hours) in three. Hematuria was noted in 11 patients in the SWL group (none required blood transfusion); while none of the patients in the RPPL had hematuria postoperatively. Urinary tract infection (UTI) occurred in 18 patients of the SWL group and in two patients of the RPPL group.

## DISCUSSION

Treatment of renal stones in the present era is one of the most fiercely discussed subjects between the treating urologists the world over. Of the various options that evolved out of the concerted efforts of the workers; open pyelolithotomy, SWL, retrograde ureterorenoscopy, with laser lithotripsy and / or basket and PCNL, have established themselves as modalities of treatment for renal pelvic stones. However, the role of retroperitoneoscopic pyelolithotomy has not been evaluated adequately, due to the inability to establish an effective pneumoretroperitoneum by direct insertion of the Veress needle. It was after the balloon dissection technique described by Gaur[2] that retroperitoneoscopic removal of renal calculi has become a viable minimally invasive option.

Even after the advent of balloon dissection techniques, retroperitoneoscopic pyelolithotomy has not gained much popularity, because of the steep learning curve, paucity of trainers, and lack of landmarks in the retroperitoneum. Even for those surgeons who perform this procedure on a regular basis, it continues to be a taxing procedure. However, the small number of centers performing this procedure has reported good success in stone clearance especially for those located in the renal pelvis. The author’s team has reported a success rate of 100% for pelvic stones, even though there was 33% failure in the case of caliceal stones.[5]

Our institution is one of the few centers performing retroperitoneoscopic pyelolithotomy in significant numbers. The role of this procedure for the management of renal calculi is well established as reported in our earlier publication.[5] The present study was conducted to compare this procedure with SWL, which is an established modality of treatment for renal stones.[12–14] SWL has been compared in the past with PCNL or retrograde intrarenal surgery, but there is no published data comparing SWL with retroperitoneoscopic pyelolithotomy.[15]

In the present study, the mean stone size in the RPPL group (1.73 cm) was larger than that in the SWL group (1.02 cm). Even though this study included a stone size of 2.5 cm or less, in our previous study RPPL was equally effective in larger stone size, including staghorn calculi.[5,16]

In our study stone clearance in RPPL was 96.5% overall, although it was 100% for pelvic stones and 62.5% for caliceal stones. Comparison with other series was not possible because of the small sample size of the other published series.[2–11] Most of the series contained less than 20 cases that should fall within the learning curve, although there was no universally accepted definition of the learning curve quantifying the number of cases [Gaur[2] (8 patients), Sinha[3] (20), Hemal[4] (7), Soares[6] (15), Kramer[7] (5), Simforoosh[8] (5), Fragoso[9] (15), Desai[10] (5), Nadu[11] (13)].

Retroperitoneal laparoscopic pyelolithotomy has a significant short coming as far as caliceal stones are concerned. Stone clearance in the inferior calyx in the hydronephrotic kidney was carried out under vision, while in the cases of middle and superior calices, it was a blind procedure resulting in a significant failure for caliceal stones.

The mean time taken to resume normal activities for RPPL was 1.09 days and the standard deviation was 0.3 day, while for SWL it was 2.37 days, with the standard deviation being 0.7 day. With the help of the Mann whitney ‘U’ test and Levene’s test the *P*- value between the two procedures was < 0.001, which was highly significant. This showed that there was a significant difference for time taken to resume normal activities between the two procedures; the time being significantly less for RPPL. The patients who underwent RPPL were up and about, performing their daily chores usually in one day’s time, whereas, patients who underwent SWL had pain after each sitting and due to multiple sittings took a longer time to resume normal activities.

The mean total requirement of analgesics for RPPL was 102.81 + 47 mg of diclofenac sodium. However, the difference was significant when compared with SWL, where the mean total requirement was 889.02 + 395.96 mg. This difference was highly significant and could be explained by the fact that SWL in our setup was done as an out patient procedure; it required multiple sittings and multiple fragments of stone that formed after each session of SWL therapy led to pain and increased analgesic requirement.

In the SWL group, average hospital visit per patient is 4.8 ± 2.8, whereas, in the RPPL group, it is 2.5 ± 0.5. It shows that the patient has to come to the hospital a fewer number of times in the RPPL group as compared to the SWL group.

The multiple sittings of shock waves given to patients of SWL also expose the patients to a greater degree of radiation. However, there is no such radiation risk during RPPL.

The cosmetic result was fairly acceptable to the patients; none of the patients complained about a small scar, ranging from 5 to 15 mm in size, present in the flank region, after RPPL.

In a study done by Eterovic *et al*.,[17] it was shown that while open pyelolithotomy, from day one, continuously improves renal function, whereas, SWL first decreases it and then over a period of months, brings it back to the pre-treatment level. These reports suggest that RPPL having procedural similarity to open pyelolithotomy, is not only nephron sparing, but also nephron reviving, and consequently, could eventually become accepted as the procedure of choice in a select group of patients with renal calculus disease.

There is a tendency among surgeons to dismiss retroperitoneoscopic pyelolithotomy as a non-viable option without adequate scientific data. In our experience this procedure has gained immense popularity among our patients, as it not only achieves complete stone clearance in one sitting, but incorporates advantages of minimal invasive surgery as well.

## CONCLUSION

Shock wave lithotripsy, being a non-invasive modality, is an established procedure for a solitary stone < 2 cm all over the world. However, RPPL achieves comparable stone clearance, fast return to normal daily activities, fewer hospital visits, and lesser analgesic requirement.
